# Evaluating the Adverse Effects and Associated Risk Factors of COVID-19 Vaccines Among Healthcare Workers: A Retrospective Study in the Duhok Province, Iraq

**DOI:** 10.7759/cureus.71671

**Published:** 2024-10-17

**Authors:** Ibrahim A Naqid

**Affiliations:** 1 Department of Biomedical Sciences, College of Medicine, University of Zakho, Zakho Duhok, IRQ

**Keywords:** covid-19 vaccines, healthcare worker, kurdistan region, risk factors, side-effect

## Abstract

Background and aim: The COVID-19 pandemic has profoundly impacted global health, necessitating the rapid development of vaccines to reduce its effects. However, concerns among healthcare workers regarding vaccine safety and side effects have led to increased hesitancy towards COVID-19 vaccination. Therefore, this study aims to assess the severity of adverse effects and associated factors of three COVID-19 vaccines among healthcare workers in Iraqi Kurdistan.

Methods: A retrospective cross-sectional study was conducted among healthcare workers in Duhok province, Kurdistan Region, Iraq, with 625 participants aged 18 to 65 years (mean age 38.42±13.96) from August to December 2022. Data were collected through face-to-face interviews. The study questionnaire consisted of two parts: the first part collected demographic information about the participants, while the second part focused on their COVID-19 infection and vaccination status.

Results: Of the total participants, 52.8% were female, with a mean age of 38.42 years (±13.96 SD). Approximately 67.5% received the Pfizer/BioNTech vaccine, and 60.9% had a prior history of COVID-19 infection. A significant proportion (82.24%) reported side effects, which were mostly mild or moderate, with 13.6% experiencing severe symptoms. The most commonly reported side effects across all three vaccines were pain at the injection site, fever, headache, and fatigue. Participants aged 36-50 reported significantly higher rates of severe side effects (87.88%, p = 0.047). Individuals with a history of allergies experienced significantly fewer adverse effects (48.93%) (p = 0.001). Those with prior COVID-19 infection also reported more severe symptoms post-vaccination (p = 0.001) and vaccine type-influenced side effects (p < 0.001), with Oxford/AstraZeneca recipients more likely to experience severe reactions compared to Pfizer recipients. Fatigue, chills, tremors, and myalgia were significantly more common in females than males (p < 0.005).

Conclusion: This study identified the most common side effects of COVID-19 vaccines among healthcare workers in Kurdistan Region of Iraq. AstraZeneca vaccine was associated with a higher prevalence of systemic effects, including fever, fatigue, headache and myalgia. These findings provide valuable insights into the safety and side effect profile of COVID-19 vaccines in the region.

## Introduction

The COVID-19 pandemic has profoundly impacted global health, including the Kurdistan Region of Iraq, necessitating the rapid development and deployment of COVID-19 vaccines to reduce its effects [1-3]. Among the frontline defenders against this virus, healthcare workers (HCWs) have borne the brunt of exposure and risk. Vaccination has emerged as a critical tool in safeguarding their health, yet it is essential to thoroughly evaluate the adverse effects associated with these vaccines, as well as the risk factors influencing these reactions. Vaccination prevents symptomatic COVID-19 infection and minimizes the risks of severe illness by stimulating the immune system to produce antibodies [4].

In response to the declaration of the pandemic, the Kurdistan Regional Government implemented a range of preventive measures to mitigate the transmission of the virus. These measures included travel restrictions, lockdowns, the suspension of public gatherings, mandatory face mask usage, and social distancing protocols [5]. Despite these efforts, COVID-19 has continued to circulate throughout the region, manifesting in various clinical presentations [5,6]. Achieving herd immunity is essential for controlling the spread of the pandemic and relies heavily on effective vaccination coverage within the population. Fortunately, the scientific community has developed several COVID-19 vaccines in a relatively short timeframe [6].

The World Health Organization (WHO) granted emergency use authorization for multiple COVID-19 vaccines. However, only three vaccines have been approved and administered in the Kurdistan Region of Iraq: the Pfizer/BioNTech mRNA vaccine (BNT162b2), the Oxford/AstraZeneca vaccine (ChAdOx1 nCoV-19), and Sinopharm (BBIBP-CorV) [7]. Unfortunately, a considerable amount of misinformation regarding various aspects of the pandemic and vaccine safety has circulated among the public [8]. This has led to significant concerns about vaccine safety and potential adverse effects, which are major barriers to vaccine acceptance [9].

Although COVID-19 vaccination is seen as the primary means of preventing the spread of the virus, it is important to recognize that no vaccine is entirely free from side effects. Individuals have reported a range of responses to vaccination, varying from minimal to severe adverse effects. Despite these potential side effects, vaccination remains an effective method of providing immunity against COVID-19 [10]. Concerns about adverse effects significantly contribute to vaccine hesitancy within the population. Addressing these concerns through enhanced public awareness of vaccine safety and efficacy, including transparency regarding potential adverse effects, is crucial [11].

This study aimed to assess the prevalence and severity of adverse effects related to COVID-19 vaccinations among healthcare workers, who are not only vital in managing patient care but also serve as a population at risk for unique vaccine responses due to their occupational exposure and underlying health conditions. Additionally, we seek to identify factors that may contribute to the occurrence of severe adverse effects. Understanding these adverse effects is crucial for informing vaccination strategies, enhancing safety protocols, and ensuring the well-being of this essential workforce.

By examining the relationship between demographic factors, occupational roles, and vaccine side effects, this research seeks to contribute to a comprehensive understanding of vaccine safety within this critical population. Ultimately, the findings aim to bolster public health efforts and improve vaccination uptake, ensuring that healthcare workers are both protected and able to continue their pivotal role in combating the pandemic.

## Materials and methods

Study design

A retrospective cross-sectional study among healthcare workers was performed in Duhok province, Kurdistan Region, Iraq with a total of 625 participants from August 2022 to December 2022. Participants ages ranged from 18 to 65 years old (38.42±13.96). The age groups were classified into four groups (18-25, 26-35, 36-50, and more than 50 years). Data acquisition occurred through face-to-face interviews conducted by the author in multiple hospitals.

According to the available data, there are 4500 registered healthcare workers in Duhok Governorate, Kurdistan Region, Iraq. The sample size was calculated using an online calculator (http://www.raosoft.com/samplesize.html) with a 95% confidence interval and 5% margin of error to be 355. The study recorded almost double this size to reduce bias and increase the reliability of the data.

Study questionnaire

The study used a modified version of a previously validated questionnaire with slight modifications to fit with the objectives of the study [12]. It included 21 items divided into two sections. The first section, with 10 items, collected demographic data like age, gender, marital status, occupation, lifestyle habits, and health history. The second section, with 11 items, focused on COVID-19 infection and vaccination status, including vaccine type, doses received, duration and severity of side effects, and any post-vaccination COVID-19 infections. Adverse effects were categorized as local or systemic, and their severity was rated on a Likert scale from 1 to 10, with a score 1-3 considered mild, 4-7 considered as moderate, and 8-10 classified as severe. The local side effects included pain, oedema, itching, redness, and hotness at the injection site. Systemic side effects included a list of symptoms such as headache, fatigue, fever, chills, joint pain, muscle pain, diarrhoea, nausea, and hair loss.

Inclusion/exclusion criteria

The inclusion criteria of the study included healthcare worker, older than 18 years old, living in the Kurdistan Region, Iraq, who had received at least one dose of three COVID-19 vaccines distributed by the Iraqi Kurdistan Ministry of Health and agreement to be recruited in the study. Exclusion criteria were applied to participants who submitted incomplete questionnaires.

Ethical approval

The research received approval from the Scientific Committee of the College of Medicine, University of Zakho, Kurdistan Region, Iraq. This approval is indicated by the letter issued with the reference number (SEP22/E11) on 06/07/2022. The questionnaires were confirmed and checked by the College of Medicine Ethics Committee, University of Zakho, Kurdistan, Iraq. Written informed consent was obtained from all participants before starting the study. The personal data and privacy of participants are also protected throughout the research process.

Statistical analysis

Statistical analysis was performed using GraphPad Prism version 8.0 (GraphPad Software, Boston, MA). Descriptive statistics were reported as frequencies and percentages as well as mean and standard deviation. The relationship between demographic characteristics and both the occurrence and severity of post-vaccination side effects was examined using the Chi-square test. A p-value of 0.05 or less was considered statistically significant.

## Results

Demographic characteristics of participants

A total of 625 participants who received the COVID-19 vaccine were recruited for the study. The mean age of the participants was 38.42±13.96 years. Females constituted 52.8% of the study population, while 33.6% were single. About 20.8% of the participants identified as smokers, and only 17.76% had chronic health problems. Only 21.28% reported having some form of allergy, with blood groups A and O being the most prevalent among the participants. Table 1 provides a summary of the demographic and health characteristics of the study respondents.

**Table 1:** Demographic characteristics of study’s vaccinated population

Characteristics	No. (%)
Age group (Year)	
Mean ± SD	38.42±13.96
18-25	125 (20)
26-35	201 (31.16)
36-50	165 (26.04)
>50	134 (21.44)
Gender	
Male	295 (47.2)
Female	330 (52.8)
Marital status	
Single	210 (33.6)
Married	415 (66.4)
Smoking	
Yes	130 (20.8)
No	495 (79.2)
Chronic health issue	
Yes	111 (17.76)
No	514 (82.24)
Have you experienced any allergies?	
Yes	133 (21.28)
No	492 (78.72)
Blood group	
A	221 (35.36)
B	118 (18.88)
O	224 (35.84)
AB	62 (9.92)

History of COVID-19 infection and vaccination data

The data presented in Table 2 provides a snapshot of COVID-19 infection and vaccination status among the participants. The majority of the participants (74.4%) had tested positive for COVID-19, with the majority receiving the Pfizer/BioNTech vaccine (67.52%), followed by Oxford/AstraZeneca (20.16%). About 80.3% of participants had received two doses, and nearly half (47.68%) were infected with COVID-19 after vaccination. A significant proportion (82.24%) reported side effects, which lasted an average of 3.1 ± 1.94 days. The severity of these adverse effects was mostly mild (35.51%) or moderate (33.12%), with 13.6% experiencing severe symptoms.

**Table 2:** History of COVID-19 infection and vaccination data among studied population

COVID-19 infection and vaccination data	No. (%)
Previously tested RTPCR test positive for COVID-19	
Yes	465 (74.4)
No	160 (25.6)
Type of vaccines	
Pfizer/BioNTech	422 (67.52)
Oxford/AstraZeneca	126 (20.16)
Sinopharm	77 (12.32)
Number of COVID-19 vaccine doses administered	
One dose	123 (19.68)
Two doses	502 (80.32)
Infected with COVID-19 after vaccination	
Yes	298 (47.68)
No	327 (52.32)
Side effect following vaccination	
Yes	514 (82.24)
No	111 (17.76)
Duration of symptoms following vaccination (Day ± SD):	3.1±1.94
Severity of side effects following vaccination:	
Mild	222 (35.51)
Moderate	207 (33.12)
Severe	85 (13.6)
No symptoms	111 (17.76)

Determinants of developing post-vaccination adverse effects among participants

Participants who had contracted COVID-19 prior to vaccination reported more severe symptoms compared to those without prior infection, and this association was statistically significant (p = 0.001). The type of vaccine administered also played a crucial role in the severity of side effects (p < 0.001). Individuals who received the Oxford/AstraZeneca vaccine were more likely to develop severe side effects compared to others. Conversely, 82.94% of those who received the Pfizer vaccine reported either no side effects or only mild ones (Table 3). The severity of side effects was also not significantly influenced by chronic health problems (p = 0.44) or smoking (p = 0.22) (Table 3). A noteworthy association was found between allergies and the severity of side effects (p = 0.001).

**Table 3:** Determinants of developing post-vaccination side-effects among demographic characteristics RTPCR: Reverse transcriptase polymerase chain reaction.

Characteristics	Yes	No	Total	P value
	n (%)	n (%)	n (%)	
Age				
18-25	102 (81.6)	23 (18.4)	125 (20.0)	0.047
26-35	166 (82.59)	35 (17.41)	201 (32.16)	
36-50	145 (87.88)	20 (12.12)	165 (26.04)	
>50	101 (73.37)	33 (24.63)	134 (21.44)	
Gender				
Male	233 (78.98)	62 (21.02)	295 (47.20)	0.06
Female	280 (84.85)	50 (15.15)	330 (52.80)	
Marital status				
Single	171 (81.43)	39 (18.57)	210 (33.60)	0.74
Married	343 (82.65)	72 (22.86)	315 (50.40)	
Smoking				
Yes	104 (80.0)	26 (20.0)	130 (20.80)	0.44
No	410 (82.83)	85 (17.17)	495 (79.20)	
Chronic health issue				
Yes	96 (86.49)	15 (13.51)	111 (17.76)	0.22
No	418 (81.32)	96 (18.68)	514 (82.24)	
Have you experienced any allergies?				
Yes	114 (48.93)	119 (51.07)	233 (37.28)	0.001
No	400 (81.30)	92 (18.70)	492 (78.72)	
Blood group				
A	177 (80.09)	44 (19.91)	221 (35.36)	0.69
B	96 (81.36)	22 (18.64)	118 (18.88)	
O	189 (84.38)	35 (15.63)	224 (35.84)	
AB	51 (82.26)	11 (17.74)	62 (9.92)	
Previously tested RTPCR test positive for COVID-19				
Yes	389 (83.66)	76 (16.34)	465 (74.40)	0.12
No	125 (78.13)	35 (21.88)	160 (25.60)	
Type of vaccines				
Pfizer	72 (17.06)	350 (82.94)	422 (67.52)	0.001
AstraZeneca	108 (85.71)	18 (14.29)	126 (20.16)	
Sinopharm	56 (72.73)	21 (27.27)	77 (12.32)	
Contracted COVID-19 post-vaccination				
Yes	265 (88.93)	33 (11.07)	298 (47.68)	0.001
No	249 (76.15)	78 (23.85)	327 (52.32)	

The severity of adverse effects according to the type of vaccines

Participants in the age group 36-50 years reported significantly higher rates of severe side effects (87.88%) (p = 0.047) (Table 4). Although females and married participants exhibited severe side effects, this difference was not statistically significant (p > 0.05). The data summarizes side effects reported after vaccination with Pfizer-BioNTech, AstraZeneca, and Sinopharm (Table 4). Fatigue was significantly more common among AstraZeneca recipients (53.2%) compared to Pfizer (40.8%) and Sinopharm (32.5%) (p = 0.008). Fever also occurred more frequently in AstraZeneca recipients (61.9%) than in Pfizer (52.8%) and Sinopharm (44.2%) (p = 0.04). Chills and tremors were higher in AstraZeneca (19.0%) compared to Sinopharm (5.2%) (p = 0.02). Diarrhea was significantly more common in Pfizer (27.7%) than in AstraZeneca (5.6%) and Sinopharm (6.5%) (p = 0.001). Nausea was reported more frequently among AstraZeneca recipients (16.7%) compared to Pfizer (7.3%) (p = 0.007).

**Table 4:** Adverse effects based on the type of COVID-19 vaccine administered

Side effects	Pfizer-BioNTech (n=422)	AstraZeneca (n=126)	Sinopharm (n=77)	Total (n=625)	P value
	n (%)	n (%)	n (%)	n (%)	
Systemic side effect					
Headache	161 (38.2)	51 (40.5)	21 (27.3)	233 (37.3)	0.136
Fatigue	172 (40.8)	67 (53.2)	25 (32.5)	264 (42.3)	0.008
Fever	223 (52.8)	78 (61.9)	34 (44.2)	335 (53.6)	0.04
Chills and tremors	60 (14.2)	24 (19.0)	4 (5.2)	88 (14.1)	0.02
Joint pain	87 (20.6)	18 (14.3)	19 (24.7)	124 (19.8)	0.15
Myalgia	116 (27.5)	39 (31.0)	14 (18.2)	169 (27.1)	0.13
Diarrhoea	117 (27.7)	7 (5.6)	5 (6.5)	129 (20.6)	0.001
Nausea	31 (7.3)	21 (16.7)	7 (9.1)	59 (9.4)	0.007
Local side effect					
Local pain	239 (56.6)	52 (41.3)	33 (42.9)	324 (51.8)	0.002
Local oedema	56 (13.3)	18 (14.3)	12 (15.6)	86 (13.8)	0.84
Itching	24 (5.7)	15 (11.9)	5 (6.5)	44 (7.1)	0.056
Local redness	41 (9.7)	13 (10.3)	4 (5.2)	58 (9.3)	0.41
Local hotness	86 (20.4)	22 (17.5)	8 (10.4)	116 (18.6)	0.11
Duration of symptoms (Day + SD)	2.91+1.93	2.75+1.81	2.88+2.28		0.78

For local side effects, local pain was reported significantly more in Pfizer recipients (56.6%) compared to AstraZeneca (41.3%) and Sinopharm (42.9%) (p = 0.002). Local edema showed no significant differences across vaccines (p = 0.84). Itching was higher in AstraZeneca (11.9%) compared to Pfizer (5.7%), but not statistically significant (p = 0.056). The average duration of symptoms was similar across all vaccine types (Pfizer: 2.91 ± 1.93 days, AstraZeneca: 2.75 ± 1.81 days, Sinopharm: 2.88 ± 2.28 days), with no significant difference (p = 0.78).

Severity of adverse effect according to gender

Fatigue was more prevalent in females (47.88%) compared to males (35.93%), with a statistically significant p-value of 0.003 (Table 4). Chills and tremors were also higher in females (17.27%) than in males (10.51%), (p=0.016). Additionally, myalgia was more common in females (30.61%) compared to males (23.05%), with a significant p-value of 0.038. Other systemic effects, such as headache, fever, joint pain, diarrhea, and nausea, did not demonstrate significant gender differences. Regarding local pain, it was more prevalent in females (59.39%) than in males (53.22%), although this difference was not statistically significant (p=0.125). Other local reactions, including oedema, itching, redness, and hotness, did not exhibit significant differences between genders (Table 5).

**Table 5:** Adverse effects of COVID-19 vaccines according to the gender

Side effects after vaccination	Male (n=295)	Female (n=330)	P value
	n (%)	n (%)	
Systemic side effect			
Headache	102 (34.58)	131 (39.70)	0.21
Fatigue	106 (35.93)	158 (47.88)	0.003
Fever	150 (50.85)	185 (56.06)	0.19
Chills and tremors	31 (10.51)	57 (17.27)	0.016
Joint pain	33 (11.19)	26 (7.88)	0.17
Myalgia	68 (23.05)	101 (30.61)	0.038
Diarrhoea	11 (3.73)	18 (5.45)	0.34
Nausea	26 (8.81)	33 (10.0)	0.68
Local side effect			
Local pain	157 (53.22)	196 (59.39)	0.125
Local oedema	39 (13.22)	47 (14.24)	0.72
Itching	18 (6.10)	26 (7.88)	0.43
Local redness	27 (9.15)	31 (9.39)	0.94
Local hotness	58 (19.66)	60 (18.18)	0.68

## Discussion

Vaccination has been fundamental in the prevention and eradication of infectious diseases. During the COVID-19 pandemic, vaccination became a cornerstone in efforts to control the virus, protect public health, and mitigate the associated economic crises. The development of effective vaccines was prioritized globally to reduce infection rates, mortality, and hospitalizations. In an exceptionally short period, several vaccines from different manufacturers became available worldwide. However, these vaccines were not without side effects, and numerous claims regarding their safety circulated within society. These misconceptions hindered the achievement of high vaccination rates [13]. In the Kurdistan Region of Iraq, limited data on the safety and side effects of COVID-19 vaccines among healthcare workers were available, creating an urgent need to address and dispel public fears. This study aims to bridge this knowledge gap by identifying the potential adverse effects and associated risk factors of the three COVID-19 vaccines available in the Kurdistan region, Iraq, thus providing better information among healthcare workers.

In the present study, around 74.4% of participants had tested positive for COVID-19, indicating a high burden of the virus among healthcare workers. This contrasts with previous studies performed in the Kurdistan Region of Iraq, which reported a lower prevalence rate (37% to 43%) of individuals with a positive history of COVID-19 infection [14,15]. The high burden of the infection observed in the present study could be attributed to its recency compared to other studies and potentially being influenced by the emergence of more COVID-19 variants and waves in the region, and it may also be because of a longer time frame. This also aligns with global trends, as many regions experienced high rates of infection prior to widespread vaccination campaigns [16]. A large portion of the vaccinated population had received the Pfizer/BioNTech vaccine (67.52%), followed by the Oxford/AstraZeneca vaccine (20.16%). This distribution reflects global vaccination trends, where mRNA vaccines like Pfizer/BioNTech were more widely distributed due to their early availability and high efficacy rates [10]. The data also show that 80.3% of participants had received two doses of a COVID-19 vaccine, consistent with recommendations from health organizations to ensure optimal immunity [17]. Despite this, nearly half of the participants contracted COVID-19 after vaccination, highlighting the challenge of breakthrough infections. While vaccines significantly reduce the risk of severe disease and death, breakthrough infections remain possible, particularly with the emergence of new variants [18]. In terms of side effects, 82.24% of participants reported experiencing post-vaccination symptoms, lasting on average 2.86 ± 1.94 days. Most side effects were mild and moderate, with a smaller percentage experiencing severe symptoms (13.6%). This is consistent with reports from clinical trials and post-marketing surveillance, which indicate that most COVID-19 vaccine side effects are mild to moderate and short-lived [19]. This is also consistent with a study from Saudi Arabia, where mild to moderate side effects were frequently reported following COVID-19 vaccination [20]. In our study, severe side effects occurred in about 13% of participants, aligning with earlier research suggesting that roughly one-tenth of vaccinated individuals may experience severe adverse reactions.

The findings of this study suggest that age may play a significant role in the severity of side effects following COVID-19 vaccination. Participants aged 36-50 years reported a notably higher rate of severe side effects (87.88%, p = 0.047). This observation aligns with other studies that have identified age as a factor influencing immune response and adverse reactions post-vaccination [20]. However, despite females and married participants showing higher occurrences of severe side effects, these differences were not statistically significant (p > 0.05). This is consistent with the previous study conducted in the United Kingdom (UK) health system, which found no strong association between gender or marital status and vaccine-related side effects [19].

Interestingly, neither chronic health conditions nor smoking habits significantly affected the severity of side effects, with p-values of 0.44 and 0.22, respectively. This lack of association contrasts with some studies that have suggested individuals with comorbidities may experience more severe side effects [12,21]. This highlights the need for further research to better understand the variability in individuals' responses and the factors that truly contribute to adverse effect severity. The significant link between allergies and severe side effects (p = 0.001) reinforces the well-documented heightened immune sensitivity in allergic individuals, who may be more prone to adverse reactions. Participants who had previously contracted COVID-19 reported more severe post-vaccination symptoms compared to those without prior infection, with this association being statistically significant (p = 0.001). This finding supports existing research that indicates prior infection may prime the immune system for a more robust response, resulting in stronger side effects. Additionally, the type of vaccine administered was found to significantly impact the severity of side effects (p < 0.001). Those who received the Oxford/AstraZeneca vaccine were more likely to report severe adverse effects, including fatigue, myalgia, fever or chills and injection side reactions such as pain, redness and swelling, while 82.94% of participants vaccinated with the Pfizer vaccine reported either mild or no side effects. This observation is consistent with prior studies comparing the side effect profiles of different COVID-19 vaccines [12,21]

The severity of adverse effects across different vaccine types, such as Pfizer-BioNTech, AstraZeneca, and Sinopharm, highlights the variability in adverse reactions reported by recipients of these vaccines. According to the data, AstraZeneca recipients experienced a higher frequency of systemic side effects compared to those receiving Pfizer or Sinopharm. For instance, fatigue was notably more common in AstraZeneca recipients (53.2%) compared to Pfizer (40.8%) and Sinopharm (32.5%), with statistical significance (p = 0.008). Similarly, fever occurred more frequently in AstraZeneca recipients (61.9%) than in those vaccinated with Pfizer (52.8%) or Sinopharm (44.2%) (p = 0.04). This trend may suggest that the viral vector-based AstraZeneca vaccine induces a more robust systemic immune response, leading to a higher incidence of side effects such as fever and fatigue [22]. Interestingly, other side effects like chills and tremors were significantly higher in AstraZeneca recipients (19.0%) compared to Sinopharm (5.2%) (p = 0.02), while diarrhea was more common in Pfizer recipients (27.7%) compared to AstraZeneca (5.6%) and Sinopharm (6.5%) (p = 0.001). These differences may reflect variations in the immune response pathways activated by the different vaccine platforms-mRNA-based vaccines like Pfizer and inactivated vaccines like Sinopharm [23].

Regarding local side effects, Pfizer recipients reported significantly more local pain (56.6%) compared to AstraZeneca (41.3%) and Sinopharm (42.9%) (p = 0.002). The local reactogenicity, which involves pain at the injection site, might be associated with the strong immunogenicity of mRNA vaccines like Pfizer, as they elicit a more localized immune response. On the other hand, local edema showed no significant differences between the three vaccines (p = 0.84), and itching was more prevalent in AstraZeneca (11.9%) compared to Pfizer (5.7%), though this result did not reach statistical significance (p = 0.056). Lastly, the duration of symptoms was comparable across all vaccine types, averaging around 2 to 3 days, with no significant differences (p = 0.78). This indicates that while the severity of specific side effects might vary depending on the vaccine, the overall duration of these side effects tends to be similar across different platforms [24].

The biological gender differences in the prevalence of post-vaccination symptoms, as highlighted in the data, are quite notable, particularly in fatigue, chills, tremors, and myalgia. The higher prevalence of fatigue in females (47.88%) compared to males (35.93%) with a statistically significant p-value (p=0.003) suggests a biological or physiological predisposition in women to experience this symptom more frequently. These could be related to hormonal differences or immune response variations between genders. It is well-documented that estrogen can influence immune system behavior, which may explain why females often report more pronounced reactions to immunizations or infections compared to males [25].

Chills and tremors being more common in females (17.27%) than in males (10.51%) (p=0.016) aligns with similar observations in other clinical settings [23,26] where women tend to report heightened or more severe systemic responses to immune challenges. Myalgia also follows this pattern (30.61% in females vs. 23.05% in males, p=0.038), further supporting the notion that females may have a more robust inflammatory response, possibly contributing to the increased prevalence of muscle pain. On the other hand, symptoms like headache, fever, joint pain, diarrhea, and nausea not demonstrating significant gender differences are interesting and may indicate that the mechanisms driving these particular reactions are not as heavily influenced by gender-related factors making it clear that the contrast is with a particular study. This contrasts with a particular study that showed significant gender differences, suggesting that different physiological pathways might be involved [25]. The finding regarding local pain, which is slightly more common in females (59.39%) compared to males (53.22%), though not statistically significant (p=0.125), could be worth further investigation. Interestingly, other local reactions such as oedema, itching, redness, and hotness not differing significantly between genders suggest that the mechanisms responsible for localized inflammatory reactions post-vaccination may be more uniform across genders [25].

Study Limitations and Strengths

Our study has several limitations. First, its focus on a single province in Iraqi Kurdistan restricts the generalizability of the findings to a national context. Second, the reliance on self-reported side effects introduces the possibility of reporting and recall bias, necessitating caution in the interpretation of the results. Finally, the study primarily addressed short-term side effects. To thoroughly investigate the long-term adverse effects associated with COVID-19 vaccines, we recommend conducting a nationwide cohort study. Our study also has some strengths. Firstly, healthcare workers are at a heightened risk of COVID-19 exposure, making them an ideal population to assess adverse effects and gain valuable insights into vaccine performance under high-exposure conditions. Secondly, healthcare workers serve as a sentinel population, identifying potential safety concerns, side effects, or risk factors early on, thus helping to guide future vaccination strategies for broader populations. Finally, this is the first study on the adverse effects of different COVID-19 vaccines among healthcare workers in the Kurdistan Region of Iraq.

## Conclusions

Participants aged 36-50 reported significantly higher rates of severe post-vaccination side effects, with allergies emerging as a notable determinant of severity. Although females and married individuals experienced more severe side effects, these differences were not statistically significant. Additionally, individuals with a history of COVID-19 prior to vaccination reported more severe symptoms, and recipients of the Oxford/AstraZeneca vaccine experienced greater side effects compared to those receiving the Pfizer vaccine. The data also revealed notable differences among vaccine recipients: AstraZeneca was associated with a higher incidence of systemic effects such as fatigue, fever, and chills, while Pfizer was linked to more local reactions, including injection site pain and diarrhea. Although local pain was more frequently reported by Pfizer recipients, local edema did not differ significantly across vaccines. Furthermore, females experienced higher rates of specific side effects, including fatigue, chills, and myalgia, compared to males, with significant differences observed in some instances. These findings suggest that gender may influence the severity of certain post-vaccination side effects.
